# Attitudes to Medication after Kidney Transplantation and Their Association with Medication Adherence and Graft Survival: A 2-Year Follow-Up Study

**DOI:** 10.1155/2014/675301

**Published:** 2014-04-28

**Authors:** Mirjam Tielen, Job van Exel, Mirjam Laging, Denise K. Beck, Roshni Khemai, Teun van Gelder, Michiel G. H. Betjes, Willem Weimar, Emma K. Massey

**Affiliations:** ^1^Department of Internal Medicine, Erasmus Medical Centre, P.O. Box 2040, 3000 CA Rotterdam, The Netherlands; ^2^Institute of Health Policy & Management (iBMG), Erasmus University Rotterdam, P.O. Box 1738, 3000 DR Rotterdam, The Netherlands; ^3^Department of Hospital Pharmacy, Erasmus Medical Centre Rotterdam, P.O. Box 2040, 3000 CA Rotterdam, The Netherlands

## Abstract

*Background*. Nonadherence to medication is a common problem after kidney transplantation. The aim of this study was to explore attitudes towards medication, adherence, and the relationship with clinical outcomes. *Method*. Kidney recipients participated in a Q-methodological study 6 weeks after transplantation. As a measure of medication adherence, respondents completed the Basel Assessment of Adherence to Immunosuppressive Medications Scale (BAASIS^©^-interview). Moreover, the intrapatient variability in the pharmacokinetics of tacrolimus was calculated, which measures stability of drug intake. Data on graft survival was retrieved from patient records up to 2 years after transplantation. *Results*. 113 renal transplant recipients (19–75 years old) participated in the study. Results revealed three attitudes towards medication adherence—attitude 1: “confident and accurate,” attitude 2: “concerned and vigilant,” and attitude 3: “appearance oriented and assertive.” We found association of attitudes with intrapatient variability in pharmacokinetics of tacrolimus, but not with self-reported nonadherence or graft survival. However, self-reported nonadherence immediately after transplantation was associated with lower two-year graft survival. *Conclusion*. These preliminary findings suggest that nonadherence shortly after kidney transplantation may be a risk factor for lower graft survival in the years to follow. The attitudes to medication were not a risk factor.

## 1. Introduction

Kidney transplant patients are required to take lifelong immunosuppressive medication to prevent graft rejection. Nonadherence to immunosuppressive medication is a common issue and increases over time. Both dosage and timing of medication are crucial. Failure to take the medication as prescribed is a risk factor for (late) acute rejection, (late) graft failure/loss, and patient mortality [1–4].

Among renal transplant patients, on average 36% of patients per year are reported to be nonadherent to immunosuppressive medication with estimates ranging from 2 to 67% [2, 5–7]. A number of patient, practitioner, and regime related factors have been shown to be related to adherence after renal transplantation. The number and frequency of medication, as well as the relationship, communication, and trust between the patient and health care provider, are likely to influence adherence [3]. Nonadherence is particularly a problem among adolescent transplant recipients. Rates of nonadherence have also been found to be related to factors such as level of social support, education, and socioeconomic status [3, 8]. There is also evidence that nonadherence prior to transplantation is an independent predictor of nonadherence after transplantation [5, 9].

As nonadherence is a behavioural rather than a medical issue, many studies have focused on exploring possible psychological and other modifiable predictors [2, 3, 10]. Psychological well-being, such as depression, can affect the extent to which an individual is adherent to the medication regime [11]. In a previous study we reported clusters of attitudes which may indicate risk of poorer adherence to medication among young adult renal transplant patients [12]. This was a population of young adults who had varying time since transplantation. Evidence suggests that adherence immediately after transplantation is often high but gradually declines over time [2], although some authors suggest that nonadherence might be “early and pervasive” among renal transplant patients [4]. Schmid-Mohler et al. [10] used the integrative model of behavioural prediction and found that forgetfulness/interruption of daily routine was the only significant predictor for nonadherence. In their later work [13] they found that nonadherence was significantly associated with patients' beliefs about their immunosuppressive medicines. The aim of this study was to gain greater insight into attitudes towards the immunosuppressive medication regime shortly after kidney transplantation. Furthermore, to explore the relationship between adherence to medication and clinical outcomes in the years following transplantation.

## 2. Methods

### 2.1. Participants

All consecutive patients who received either a living or deceased donor kidney transplant in the Erasmus Medical Centre, Rotterdam, between August, 2010, and October, 2011, were invited to participate in the study. The inclusion criteria required that kidney transplant patients were older than 18 years, had a functioning graft six weeks after transplantion, and had a sufficient level of understanding and speaking of the Dutch language. For clinical endpoints we had a follow-up time of at least two years after transplantation (until October 31, 2013).

All participants provided written consent for participation and the study was approved by the Medical Ethics Committee of the Erasmus Medical Centre.

### 2.2. Measures and Procedure

To explore attitudes towards medication after kidney transplantation we used Q-methodology. This is a method that combines aspects of qualitative and quantitative methods and provides a foundation for the systematic study of subjectivity (e.g., peoples' viewpoints or beliefs and in this case attitudes to the immunosuppressive medication regime after kidney transplantation) [14, 15]. The results of a Q-methodological study can be used to describe a population of viewpoints, not a population of people [16, 17]. In previous studies we generated statements for young adults and the elderly using the WHO dimensions of adherence [8, 18]: socioeconomic related factors, health care team or health system related factors, condition related factors, treatment related factors, and patient related factors. This was done based on an iterative procedure and consensus [12]. For the current study the statements were tailored for a more general use with patients of all ages. The final Q-set consisted of 37 statements (Table 1), which were randomly numbered and printed on cards.

**Figure 1:**
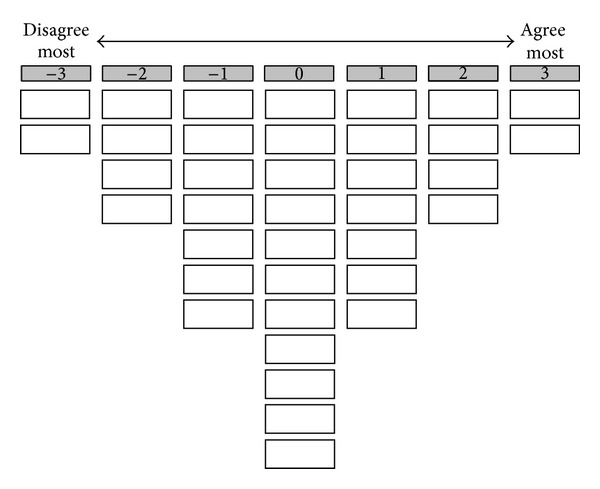
The grid that patients used to rank-order the 37 statements.

Respondents were invited to participate in face-to-face interviews. Patients were interviewed 6 weeks after transplantation during which they were asked to rank-order the 37 statements, using a quasinormal grid ranging from −3 to +3 (Figure 1) [12]. In addition, participants were asked to explain the ranking of the 2 statements that they agreed with (+3) and disagreed (−3) the most. The individual rankings of statements were analysed using by person factor analysis so as to reveal a limited number of corresponding patterns in the way the statements were sorted by respondents. Correlation between individual rankings of statements is viewed as an indication of similarity in attitude.

The outcome variable was nonadherence. To study nonadherence effectively we used a combination of measurement methods, as proposed by Farmer [19]. Firstly, we used the Basel Assessment of Adherence to Immunosuppressive Medications Scale (BAASIS^©^-interview) [20, 21]. This scale is a self-report instrument that consists of 4 questions on the taking and timing of medication, drug holidays, reduction of the dose, and persistence over the past month (Table 2). An affirmative answer to any of the first 4 questions results in assignment to the nonadherent group. This scoring is intentionally strict due to an assumption of the underreporting of nonadherence. Patients also rated their own adherence using a visual analogue scale from 0% (medication never taken as prescribed) to 100% (medication always taken as prescribed). The BAASIS measure was selected as it is short, reliable, valid, and sensitive to both timing and taking which is of particular importance for the immunosuppressive regime after kidney transplantation [20]. A number of studies have demonstrated support for the validity of both parts of the instrument [22, 23]. Specificity and sensitivity of the visual analogue scale have been shown to be high [22].

Secondly, we calculated patient intraindividual variability in the pharmacokinetics of immunosuppressive medication, in this case tacrolimus (Prograft) [24]. Whole blood tacrolimus concentrations in different measurements over time within patients were used to calculate intraindividual variability. Patients with a high intrapatient variability have tacrolimus concentrations that are often outside the therapeutic window. Underexposure may lead to immune activation, and overexposure can result in CNI-induced nephrotoxicity. Both could affect long-term outcome. Borra et al. [24] showed that high intraindividual variability in the pharmacokinetics of tacrolimus leads to reduced graft survival. One of the most likely causes for intrapatient variability is medication nonadherence. To calculate the intrapatient variability in tacrolimus concentrations we used the method previously described by Borra et al. [24].

For the clinical endpoints, we collected information about rejection (yes/no) and graft failure (yes/no) two years after transplantation.

### 2.3. Statistical Analysis

Independent *t*-tests and chi-squared analyses were conducted to test differences between responders and nonresponders. As the BAASIS overall rating scale was negatively skewed, a Mann-Whitney test was used to test the difference on this scale between adherent and nonadherent patients. One-way ANOVA and chi-squared tests were used to test the association between attitudes and adherence. When cell values were small, Fisher's exact tests were used to test 2 × 2 associations. Survival analyses were calculated with Kaplan-Meier and life table. Analyses were carried out using the Statistical Package for Social Sciences, version 20.0. Q-methodological data were analysed using PQMethod 2.11 developed by Schmolck and Atkinson 2002.

## 3. Results

### 3.1. Demographic Characteristics

Between August, 2010, and October, 2011, 212 kidney transplantations were carried out in our centre. Of these 212 patients 44 were excluded for the following reasons: inability to speak the Dutch language sufficiently (*n* = 23), a mental or physical disability (*n* = 9), death prior to inclusion (*n* = 4), graft loss (*n* = 4), primary nonfunction (*n* = 3), and follow-up at another centre (*n* = 1). Of the 168 kidney patients who were eligible to participate, 113 patients were included (67.3%). Fifty-five kidney transplant patients (32.7%) did not want to participate because they were not interested (*n* = 20) or did not want to stay longer at the outpatient clinic for the study (*n* = 26). Seven did not want to participate for logistical reasons and 2 were discontent with their treatment and decided not to participate. Demographic characteristics of respondents and nonrespondents are shown in Table 3. Of the 113 participants we had a minimum follow-up of two years; 35 experienced graft rejection and 5 graft failures (1 unknown and 4 due to rejection) and 6 patients died with a functioning graft.

### 3.2. Attitudes

The analysis of the Q-methodological study revealed three distinct attitudes towards medication adherence (Table 1). Of the 113 participants 23 did not load significantly on any of these attitudes or on more than one. Of the remaining 90 participants, 40 patients defined factor 1, 38 factor 2, and 12 factor 3.

Patients defining the first factor find it important to take their medication exactly every twelve hours (statement 29). They take good care of their kidney (statement 20) and have no worries about the future (statement 9) and are not afraid they have to go on dialysis again (statement 10). They find it reassuring that their kidney function is checked regularly (statement 34); these patients feel the least gloomy or depressed (statement 13). They do not mind taking multiple medicines every day (statement 21) and also indicate not experiencing many side effects (statement 14). This factor was labeled “confident and accurate.” These quotes from participants defining this factor illustrate this attitude profile: “*this kidney was given to me by my wife; I have an obligation to take good care of this kidney*”; “*You do not have any influence on things going wrong; I will do the best I can”.*

Patients defining the second factor also found it reassuring that their kidney function is checked regularly (statement 34), but this is more out of fear of graft loss. They are concerned that their kidney will be rejected (statement 8) and are afraid to go (back) on dialysis (statement 10). Therefore they are careful and they do not think it is wise to forget medication, even if it is only now and then (statement 2). They would rather be adherent than to enjoy their life to the fullest (statement 3). This factor was labeled “concerned and vigilant.” These quotes illustrate this attitude: “*I'm always so worried; after my check-up I always call my doctor for the test results”; “Rejection is always on my mind; this has an impact on my life”; “It is so important to stay focused on the regime; I do not want to blame myself for ruining this kidney; you have to follow the rules*”.

Patients defining the third factor find their appearance important (statement 11) and are afraid that the medication will influence their appearance negatively (statement 12). They do not want their lives to revolve around their disease (statement 5), although they indicate experiencing side effects (statement 14). Nevertheless they do not feel the need to be extra careful with their kidney from a loved one (statement 20), and they are not really concerned that they will have to go (back) on dialysis (statement 10). They want their own say in their treatment (statement 35) and feel they are able to manage their medication and appointments themselves (statement 15). Therefore this factor was labeled “appearance oriented and assertive.” These quotes illustrate this attitude:* “I do not feel sick; not everybody knows I have a kidney transplant”; “I have been a kidney patient for 40 years now, and I want to be involved”; “In the future I want to do things without thinking about my disease”; “This kidney is from my mum and that is special to me, but I am not extra careful with my kidney because it is from my mum”.*

There was general consensus between participants regarding a number of statements. In none of the attitudes patients were ashamed of their transplantation, minded others knowing about their kidney transplant (statement 1), or experienced problems with swallowing larger pills (statement 23). All attitudes were neutral about having a healthy lifestyle (statement 7), taking their medication with them when they go out of the house (statement 30) and letting the doctor know if they took a wrong dose of the medication (statement 37).

### 3.3. Adherence

The BAASIS-interview revealed that six weeks after transplantation, 17% (*n* = 19) were classified as nonadherent (missed a dose or >2 hours earlier or later than prescribed in the past 4 weeks). Nine patients (8%) had missed a dose in the last month. Twelve patients (11%) had taken their dose 2 hours before or after the prescribed time; and two patients had either missed a dose or taken their dose 2 hours before or after the prescribed time. None of the patients had altered their dose or completely stopped taking their medication in the past four weeks (Table 2). Demographic characteristics of the self-reported nonadherent patients versus the self-reported adherent patients are shown in Table 4. There were no significant differences in age, gender, education level, donor kidney, ethnicity, or social status between these groups.

Patients also rated their own overall adherence from 0 to 100%. A Mann-Whitney test revealed a significant difference between groups: the adherent group had a median of 100% and the nonadherent group had a significantly lower median of 95% (*P* < 0.01).

Of the 19 patients who were classified as nonadherent, 8 patients loaded on attitude 1, 4 patients on attitude 2, 2 patients on attitude 3, and 5 patients did not load on any specific attitude. There was no significant association between attitudes and self-reported nonadherence classification (*χ* ^2^(2) = 1.344, *P* = 0.476).

In order to calculate the intrapatient variability in tacrolimus we used a minimum of 3 tacrolimus measurements per patient (*n* = 4) and a maximum of 5 measurements per patient (*n* = 87). For 7 patients we were not able to calculate the intrapatient variability because of missing data. The median intrapatient variability was 14.5% (range of 1.12–86.3%). As a cut-off we divided the group in tertiles and split the patients into a group with low intrapatient variability (the patients in the lowest tertile, 0–11.7%) and a group with high variability (the patients in the highest tertile, 18.02–100%). This resulted in 34 patients in the low-variability group, with a mean variability of 8.9%, and 35 patients with high variability, with a mean variability of 27.0%. The intrapatient variability was significantly correlated with attitude profile (*χ* ^2^(2) = 6.799; *P* = 0.036). Patients with a high variability loaded more often on the attitude “concerned and vigilant,” while those with a low variability loaded more often on the attitude “confident and accurate”. Intrapatient variability was not correlated with the BAASIS classification of adherent versus nonadherent patients (*χ* ^2^(1) = 2.88, *P* = 0.110).

### 3.4. Clinical Endpoints

**Figure 2:**
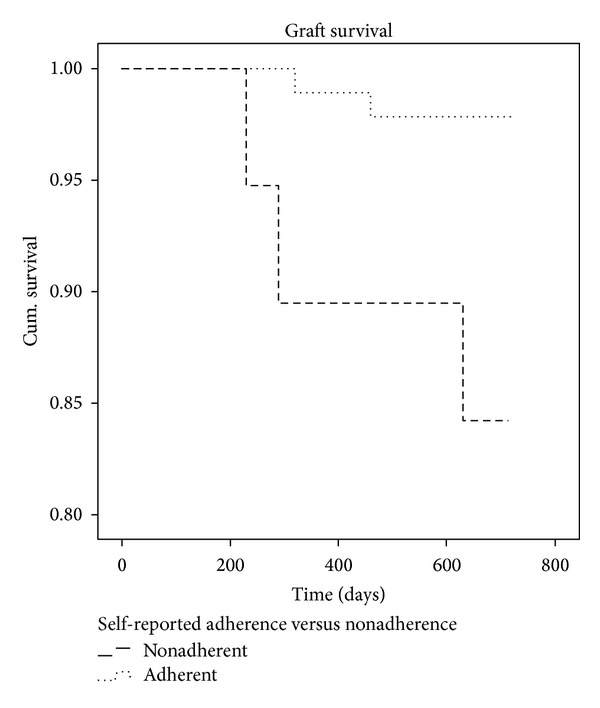
Kaplan-Meier Graft survival. The nonadherent group consisted of 19 patients (3 graft failures) and the adherent group consisted of 94 patients (2 graft failures).

Patients that reported nonadherence in the BAASIS^©^-interview (*n* = 19) had a lower two-year graft survival (failure *n* = 3) compared to the adherent group (failure *n* = 2) (84% versus 98%, resp.) (*χ* ^2^(1) = 6.409; *P* = 0.038). See Figure 2. Graft failure was not related to attitudes (*P* = 0.532) or intrapatient variability (*P* = 0.159). Patients with rejection (*n* = 35) had no significantly lower graft survival (*P* = 0.167) and graft rejection was not correlated with self-reported adherence (*χ* ^2^(1) = 0.004; *P* = 0.574), the three attitudes (*χ* ^2^(2) = 2.391; *P* = 0.347), or intrapatient variability (*χ* ^2^(1) = 2.947; *P* = 0.074).

## 4. Discussion

This Q-methodological study revealed three distinct attitudes toward medication nonadherence as early as six weeks after transplantation: (1) confident and accurate, (2) concerned and vigilant, and (3) appearance oriented and assertive. We observed association between these attitudes, but not with self-reported adherence and clinical outcomes 2 years after transplantation. Patients with the attitude “confident and accurate” appeared not to have no problems with medication adherence. They were confident about managing their medication regime 6 weeks after transplantation and these patients had significantly lower variability of tacrolimus in their blood. Earlier research has suggested that lower variability indicates greater adherence [24]. In the attitude “concerned and vigilant” we found evidence for a relationship between anxiety about the medication regime and nonadherence, as indicated by the significantly higher variability of tacrolimus in the blood of these patients. DiMatteo et al. [25] also found a difference in risk of nonadherence between anxious and nonanxious patients. They argued that patients were worried about their future and that this translated into frequent monitoring, fear of forgetting, and noncompliance. In our study, significantly more patients with a high intrapatient variability loaded on this “concerned and vigilant” attitude. These findings suggest that individuals characterised by anxiety about their medication regime may be less adherent or, alternatively, that patients who have more problems with their medication are more worried. This association between attitude and adherence was, however, only found when adherence was measured using levels of medication found in the blood, not when using self-report. Although the patients defining attitude 2 seem to indicate they are reliable in their medication taking, the high intra-patient variability in the pharmacokinetics of tacrolimus suggests differently. This discrepancy suggests a potential underreporting of nonadherence in this group on the self-report measure. Several studies before have found differences between self-reported nonadherence, which is simpler to measure but more susceptible to error, and direct measures for defining nonadherence such as drug levels or a clinical indicator, which are more objective and accurate but expensive [19, 26, 27]. Patients with the attitude “appearance oriented and assertive” want to live a normal life, be in control, and think they are capable of taking care of their kidney, and they are also the patients that indicate experiencing side effects. Although their intrapatient variability of tacrolimus was not elevated, we speculate that these patients may have a higher risk of nonadherence in the future because they are concerned about (cosmetic) side effects of their medication regimen.

The Q-methodology study thus uncovered different attitudes towards medication adherence, which were associated with intrapatient variability of tacrolimus but not with self-reported nonadherence and with clinical endpoints such as graft rejection or graft survival. In contrast, self-reported nonadherence 6 weeks after transplantation was associated with graft failure in the subsequent 2-year period, but variability of tacrolimus was not.

The findings reported should be interpreted in the light of a number of limitations. Firstly, as discussed, there is a possibility of underreporting nonadherence given the clinical setting of the study and the possibility of socially desirable answers. The BAASIS scale was developed with this phenomenon in mind and is therefore intentionally strict in its scoring mechanism: even a small deviation in the regime leads to being classified as nonadherent [20]. However, these scores were not associated with intrapatient variability of tacrolimus, a direct and potentially more objective measure for adherence, so that underreporting cannot be dismissed. Secondly, patients who did not have sufficient mastery of the Dutch language were excluded and another group of patients declined to participate. It is possible that these harder-to-reach patients demonstrate yet another attitude, not identified here. Findings therefore cannot be generalised to these patients. Finally, this study was conducted among a limited number of patients in a single centre. Replication of this study in different centres, with particular attention to inclusion of the harder-to-reach patients as well, is therefore advised.

There are a few clinical implications of these findings. We found that Q-methodology was a useful tool for nurses in their interactions with patients, as it helped patients to talk freely about a difficult clinical topic. This approach offered patients the opportunity to (visually) structure their thoughts and nurse researchers the opportunity to investigate such pertinent issues in greater depth and to develop and tailor education programmes for this patient population. In any case, the finding that self-reported nonadherence was related to likelihood of graft failure suggests that a dialog between nurse and patient on medication adherence early in the transplant recovery period could be a useful tool to flag up individuals at risk of graft failure. Future research is also needed to further explore the (reciprocal) relationship between worry/anxiety and nonadherence and its clinical consequences.

## Figures and Tables

**Table 1:** Statements and factor scores.

Statements	Posttransplant attitudes		
	Factor 1	Factor 2	Factor 3
(1) I would rather not tell others that I have a transplant	−2	−2	−3
(2) If you forget your medication now and then, it is not a problem	−1	−3**	−1
(3) It is more important to enjoy life than to be compliant	0**	−2*	−1*
(4) If I do something that is not so healthy, I tend to feel guilty	−1**	0	0
(5) I do not want my life to revolve around my disease	+1*	+2*	+3**
(6) I do not like to take medication when others are around	−1**	−2**	0**
(7) I have a healthy lifestyle	0	0	0
(8) I am worried that my kidney will be rejected	0*	+3**	0*
(9) I am concerned about my future	−3**	0**	−1**
(10) I am scared I will have to go on dialysis (again)	−1**	+2**	−3**
(11) My appearance is not very important to me	0**	−1**	−2**
(12) I struggle with the fact that my medication makes me fatter	−1**	0**	+1**
(13) I often feel gloomy and depressed	−3**	−1**	−1**
(14) I have side effects from my medication	−1**	0**	+2**
(15) I can manage my own medication and appointments	+1**	+1**	+3**
(16) My loved ones interfere too much with my health	−1	−1	−1*
(17) I receive enough support from friends and/or family	+2	+1**	+2
(18) I would like to meet other kidney transplant patients	0	−1	0**
(19) I appreciate it when others remind me to take my medication	0	0**	0
(20) I am extra careful with this kidney because it is from a loved one	+2**	+2**	−1**
(21) I do not mind taking multiple medications a day	+1**	0	0
(22) When I sleep in, I just take my medication later	0**	−1**	−2**
(23) I have problems swallowing larger pills	−2	−1	−1
(24) I sometimes forget my medication	−2	−3*	−2
(25) I know what my medications do	+1	0	+1
(26) I have a regular daily routine	0	0	0*
(27) A pillbox is a handy aid	+2	0**	+1
(28) I want the medication to stay the same if I feel good	0	−1**	0
(29) I take my immunosuppressive drugs exactly every twelve hours	+3**	+1	+1
(30) I find it reassuring to have my medication with me when I am away from home	0	+1	+1
(31) If I am not sure whether I have already taken my pill, I just take it again	−2	−2	−2
(32) The doctors know what is best for me	+1**	+1	0
(33) If I do not comply with the regime; it is ok for healthcare professionals to confront me with the consequences	+2	+2	+1*
(34) I find it reassuring that they check my kidney functioning regularly at the outpatient clinic	+3*	+3	+2
(35) I like it when the doctor gives me a say in the treatment	+1*	+1*	+2*
(36) I am honest with the doctor about my medication intake	+1	+1**	+1
(37) If I take a wrong dose of medication, I let my doctor know	0	0	0

Note: *P < 0.05;**P < 0.01. A “−3” score indicates that a typical kidney transplant patient with that posttransplant attitude would disagree most with that statement and a “+3” score that (s)he would agree most.

**Table 2:** Adherence 6 weeks after transplantation as measured with the BAASIS^©^-interview^a^ (*n* = 113).

Part 1^b^		Response *n* (%)	Categorized as nonadherent *n* (%)
1a	Taking dimension: Do you remember missing a dose of your immunosuppressive medication (IM) in the past 4 weeks?		9 (8.0)
	(i) Once	8 (7.1)	
	(ii) 2-3 times	1 (0.9)	
1b	Drug holidays: Do you remember having skipped two or more doses of your IM in a row in the past 4 weeks?	0	
2	Timing dimension: Do you remember having taken your IM more than 2 h before or after the prescribed dosing time in the past 4 weeks?		12 (10.6)
	(i) Once	10 (8.8)	
	(ii) 2-3 times	1 (0.9)	
	(ii) 4-5 times	1 (0.9)	
3	Reduction of dose: Have you altered the prescribed amount of your IM during the past 4 weeks without your doctor telling you to do so?	0	
4	Persistence: Have you stopped taking your IM completely in the past 4 weeks without your doctor telling you to do so?	0	
Total			**19 (16.8)**

Part 2^c^		Median (range)	

5	Overall adherence rating	100 (77–100)	

Note: ^a^©University of Basel, Leuven-Basel Adherence Research Group, Institute of Nursing Science, University of Basel, Belgium, 2005. Permission and conditions to use the BAASIS can be obtained from sabina.degeest@unibas.ch. ^b^Response categories for questions 1 to 4 are given on a 6-point scale: (0) no, (1) once, (2) 2-3 times, (3) 4-5 times, (4) every 2-3 days, and (5) almost daily. ^c^visual analogue scale ranging from 0% (medication never taken as prescribed) to 100% (medication always taken as prescribed).

**Table 3:** Patient characteristics of respondents and nonrespondents.

Demographics	Respondents (*n* = 113)	Nonrespondents (*n* = 55)	*P* value
	*n* (%)	*n* (%)	
Age (years)			
18–29	9 (8%)	5 (9.1%)	0.804
30–45	27 (23.9%)	8 (14.5%)	0.161
46–64	59 (52.2%)	34 (61.8%)	0.240
65+	18 (15.9%)	8 (14.5%)	0.816
Gender			
Male	73 (64.6%)	37 (67.3%)	0.733
Female	40 (35.4%)	18 (32.7%)	
Education level			
High	22 (19.5%)	5 (10%)	0.134
Middle	64 (56.6%)	28 (56%)	0.940
Low	27 (23.9%)	17 (34%)	0.180
Unknown		5	
Ethnicity			
Caucasian	86 (78.9%)	40 (74.1%)	0.489
Asian	9 (8.3%)	4 (7.4%)	0.851
African	10 (9.2%)	4 (7.4%)	0.705
Turkish	2 (1.8%)	3 (5.6%)	0.195
Other	2 (1.8%)	3 (5.6%)	0.195
Unknown	4	1	
Kidney transplant			
Living donor	89 (78.8%)	42 (76.4%)	0.725
Deceased donor	24 (21.2%)	13 (23.6%)	
Number of transplants (median, range)	1 (1–5)	1 (1–3)	0.690
Marital status			
Married	69 (63.3%)	28 (51.9%)	0.161
Living together	14 (12.8%)	5 (9.3%)	0.502
Single	16 (14.7%)	19 (35.2%)	**0.003**
Divorced	8 (7.3%)	1 (1.9%)	0.149
Widow/widower	2 (1.8%)	1 (1.9%)	0.994
Other	4	1	

**Table 4:** Demographics of self-reported nonadherent versus adherent patients.

Demographics	Nonadherent (*n* = 19)	Adherent (*n* = 94)	*P* value
	*n* (%)	*n* (%)	
Age (years)			
18–29	2 (10.5%)	7 (7.4%)	0.651
30–45	3 (15.8%)	24 (25.5%)	0.364
46–64	11 (57.9%)	48 (51.1%)	0.587
65+	3 (15.8%)	15 (16%)	0.985
Gender			
Male	11(57.9%)	62 (66%)	0.503
Female	8 (42.1%)	32 (34%)	
Education level			
High	2 (10.5%)	20 (21.3%)	0.280
Middle	12 (63.2%)	52 (55.3%)	0.529
Low	5 (26.3%)	22 (23.4%)	0.786
Ethnicity			
Caucasian	15 (78.9%)	71 (78.9%)	0.995
Asian	0 (0%)	9 (10%)	0.150
African	3 (15.8%)	7 (7.8%)	0.272
Turkish	0 (0%)	2 (2.2%)	0.512
Other	1 (5.3%)	1 (1.1%)	0.220
Unknown	0	4	
Kidney transplant			
Living donor	16 (84.2%)	73 (77.7%)	0.524
Deceased donor	3 (15.8%)	21 (22.3%)	
Number of transplant (median, range)	1 (1-2)	1 (1–5)	1.000
Marital status			
Married	12 (63.2%)	57 (60.6%)	0.837
Living together	0 (0%)	14 (14.9%)	0.072
Single	4 (21.1%)	12 (12.8%)	0.345
Divorced	1 (5.3%)	7 (7.4%)	0.735
Widow/widower	1 (5.3%)	1 (1.1%)	0.205
Other	1 (5.3%)	3 (3.2%)	0.656
